# The Attitude of Great Writers towards Disease

**Published:** 1892-07-23

**Authors:** 


### The Attitude of Great Writers Towards Disease.

### VI.—ROBERT BURTON.

Melancholy, in the wide meaning assigned to it by Burton, is a form of suffering which lends itself to any age, and while posing as a novelty to each succeeding generation, remains for ever substantially the same. The anatomy of melancholy is singularly little out of date. Many matters at which Burton glances in one of his favourite "digressions," with wistful agnosticism—problems of geography, astronomy, or surgery—are now lifted out of the region of romantic speculation. Some abuses have been rectified; some dreams realised. But, spite of the changes wrought by science and art, the prevailing complaints, vices, and aspirations of our own time seem as his pages are scanned, to be but reflections of those of our ancestors three hundred and fifty years ago, and the hysteria, depression, and innumerable minor forms of madness, which it is the fashion to ascribe to our modern hurry of living, Burton shows affecting mankind in full force almost since the world began. "For, indeed, who is not a fool, melancholy, mad? Ask not with him in the play, 'What madnesse ghosts this old man,' but what madnesse ghosts us all? For we are all mad, not once, but always so, ever and altogether as bad as he." Are we any saner yet?

Burton will show that the disease extends not only to the lower animals, but also to "vegetals and sensibles." We may smile at his pretty instance of love-melancholy, but has not Darwin taught us more romantic things even than this? "Some plants will be sick for love; ready to dye and pine away; which the husbandmen perceiving, saith Constantine, stroke many palms that grow together, and so stroking again the palm that is enamoured, they carry kisses from one to the other." And again, "Of all other, dogs are most subject to this malady, in so much some hold they dream as men do, and through violence of melancholy run mad." But the melancholy dog is nothing to the sulky alligator who has fasted eighteen months to show his dislike to "spring cleans."

There is hardly a form or cause of the disease enumerated by Burton which has not its parallel in modern times. Has not Mr. Rudyard Kipling brought up to date in his "Mark of the Beast," the fabled Leucanthropia, "which troubles men most in Februarie—they lye hid most part of the day, and go abroad in the night, barking, howling at graves and deserts"? And if Burton has a "Digression of the nature of Spirits, bad Angels, or Devils, and how they cause Melancholy," are not our ears yet ringing with the exorcism of the little boy who would not go to church? The patient who "could make Latine verses when the moon was combuste—otherwise illiterate," savours of thought reading. And if any "will attribute no vertue at all to the Heavens, or to the Sun or Moon more than he doth to their signes at an Innekeeper's post; I refer him to" Miss Rosa Baughan, of the *Lady's Pictorial,* to the Chiromancer's Association, the *Astrologer's Gazette,* or *Old Moore's Almanac.*

Poverty and want, too, are causes which are never out of fashion, nor "an heap of other accidents causing melancholy" too numerous even to mention.

Then as now, much stress was laid on diet, but on this point we recognise that views have changed. Burton does not leave much choice to the melancholy. "Beef, a strong and hearty meat, is condemned by Galen to breed gross, melancholy blood. Pork naught for greasie stomachs. Hart and red deer hath an evil name; a strong and great-grained meat, next unto a horse. All venison is melancholy and begets bad blood," yet "a pleasant meat in great esteem with us in our solemn feasts. Hare, a black meat, melancholy and hard of digestion; it breeds incubus often eaten, and causeth fearful dreams." And so on with nearly every kind of fish, flesh, or fowl. But, moreover, "milk, and all that comes of milk, as butter, cheese, curds, &c., increase melancholy." "Amongst herbs to be eaten I finde gourds, cowcumbers, coleworts, melons disallowed, but especially cabbage. It causeth troublesome dreams, and sends up black vapours to the brain." This recalls the excuse of the Scotch farmer who was pressed by the Duchess of Buccleugh at a tenant dinner to add greens to his boiled beef, "Your Grace maun alloo its a vary windy vegetable."

But, indeed, there is but little left when the Index Expugatorius is complete. "Roots are windy and bad—make men mad, especially garlick, Onyons, if a man liberally feed on them a year together." (This is credible.) "Crato utterly forbids all manner of fruits. All Pulse are naught, Beans, Peas, Fitches, &c. Bread that is of baser grain, or overhard-baked, crusty and black (let Mr. Payn's correspondents in the *Illustrated* beware), is often spoken against as causing melancholy juyce, and wind."

So now there is nothing but drink left, and here Burton, apparently a total abstainer ("I drink none myself"), is singularly unprejudiced. The pros and cons are set forth with entire impartiality, and excess only condemned; nevertheless, "the thinnest, whitest, smallest wine is best."

We get a curious mixed judgment on tobacco, "divine, rare, super-excellent tobacco, a sovereign remedy to all diseases," but then a few lines lower, "a plague, a mischief, develish and damned tobacco, the ruin and overthrow of body and soul."

The sports by which men sought to banish melancholy were much the same then as now. Burton reviews them all. "Chesse playe is a good and witty exercise of the mind, and fit for such melancholy as are idle and have extravagant impertinent thoughts," but "for some men's brains too full of anxiety, all out as bad as study; besides, it is a testy cholerick game, and very offensive to him that loseth the mate." It is no surprise in those early Puritan days to read, "Many too nicely take exception at Cardes, Tables, and Dice, and such mixt lusorious lots."

He has the modern theories about change of air. "He that loves his health must often shift places, and make choice of such as are wholesome, pleasant, and convenient." "Great heed is to be taken how we let in or exclude this ambient aire." Our ancestors seem to have excelled in excluding rather than in letting it in. "Montanus will have his patient not open a casement in bad weather, or in a boisterous season; he specially forbids us to open a window to a south wind."

"Of colours it is good to behold green, red, yellow, and white, and by all means to have light enough, with windows in the day, wax candles in the night, neat chambers, good fires in winter, merry companions." What is this but Mrs. Battle's "clear fire, clean hearth, and the rigour of the game"?

Leaving these natural alleviations we "come now at last to Pharmaceutice, or that kind of Physick which cureth by medicines." "The Divell himself was the first inventor of it. *Inventum est medecina meum,* said Apollo, and what was Apollo but the Divell?" This is manifestly unfair. But wait awhile and our author will cheerfully refute himself. "I acknowledge it a most noble and divine science, in so much that Apollo, Æsculapius and the first founders of it, were worthily accounted gods."

Many and curious are the prescriptions; one must suffice. "'Tis not amiss to bore the skull with an instrument to let out fuliginous vapours." Who would not welcome such a chimney to the brain on one of these July days when the mental smoke hangs thick and heavy in the head? Yet perhaps with Alexander Messaria we must conclude, "'tis too stiffe an humour and too thicke to be so evaporated."

